# Developmental stages of the blue‐breasted quail (*Coturnix chinensis*)

**DOI:** 10.1111/asj.13119

**Published:** 2018-10-28

**Authors:** Yoshiaki Nakamura, Yoshifumi Nakane, Masaoki Tsudzuki

**Affiliations:** ^1^ Laboratory of Animal Breeding and Genetics Graduate School of Biosphere Science Hiroshima University Higashi‐Hiroshima Hiroshima Japan; ^2^ Japanese Avian Bioresource Project Research Center Hiroshima University Higashi‐Hiroshima Hiroshima Japan; ^3^ Institute of Laboratory Animals Graduate School of Medicine Kyoto University Sakyou Kyoto Japan

**Keywords:** avian embryonic development, blue‐breasted quail, galliformes, staging table

## Abstract

Chickens and Japanese quail (*Coturnix japonica*) have traditionally been the primary avian models in developmental biology research. Recently, the blue‐breasted quail (*Coturnix chinesis*), the smallest species in the order Galliformes, has been proposed as an excellent candidate model in avian developmental studies owing to its precocious and prolific properties. However, data on the embryonic development of blue‐breasted quail are scarce. Here, we developed a normal developmental series for the blue‐breasted quail based on developmental features. The blue‐breasted quail embryos take 17 days to reach the hatching period at 37.7°C. We documented specific periods of incubation in which significant development occurred, and created a 39‐stage developmental series. The developmental series for the blue‐breasted quail was almost identical to that for chickens and Japanese quail in the earlier stages of development (stages 1–16). Our staging series is especially useful at later stages of development (stages 34–39) of blue‐breasted quail embryos as a major criterion of staging in this phase of development was the weight of embryos and the length of third toes.

## 1 INTRODUCTION

Avian embryos have been commonly used in the field of developmental biology to study developmental processes and cell fate within the embryo. Unlike mammalian embryos, avian embryos develop outside the mother within eggs, which enables access to and manipulation of the developing embryos. Moreover, fertilized avian eggs can be stored at 15–20°C for several days and then placed in an incubator to easily obtain embryos at a specific developmental stage.

**Figure 1:**
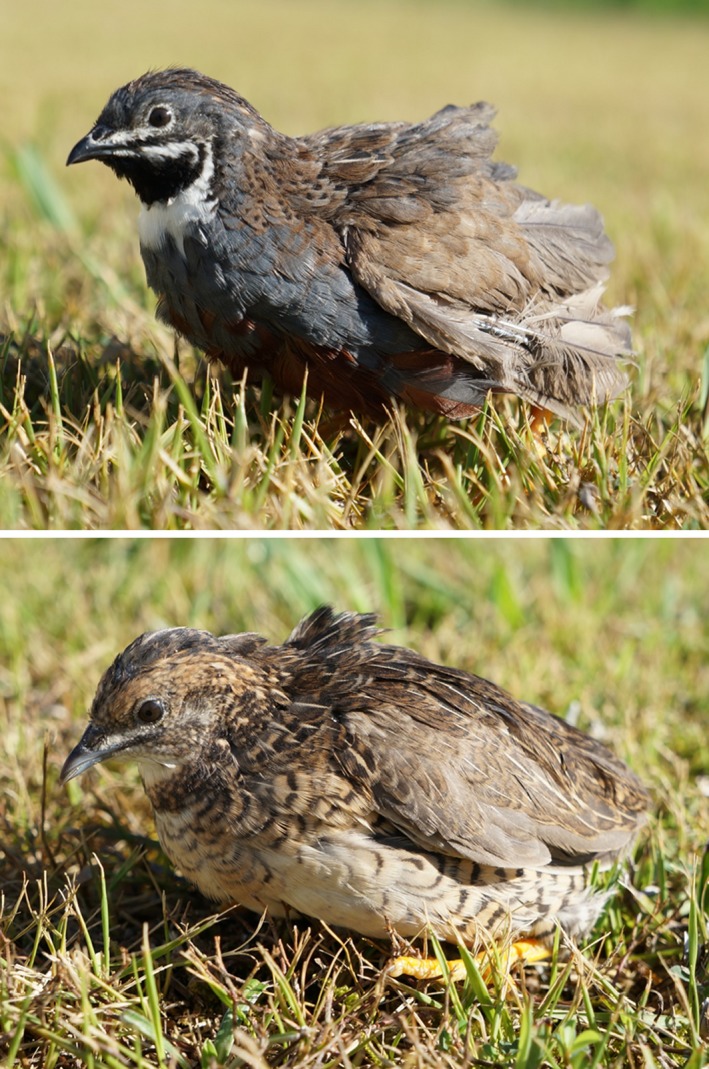
Images of adult blue‐breasted quail. The upper and lower panel show male and female blue‐breasted quail, respectively

Chickens have been traditionally utilized as the primary model in avian developmental studies. The Japanese quail (*Coturnix japonica*) has also been widely used for developmental studies owing to its many advantages, for example, small body size, hardiness, short generation interval, and prolificity. Therefore, our current understanding of avian development is primarily based on studies using chicken and Japanese quail embryos. However, the field of developmental biology has always used an extensive variety of animal models. Over the last two decades, the blue‐breasted quail (*Coturnix chinensis*; Figure 1) has been identified as an excellent candidate avian research model, equal or superior to the Japanese quail (Kageyama, Takenouchi, Kinoshita, Nakamura, & Tsudzuki, 2018; Ono et al., 2005; Parker et al., 2010; Tsudzuki, 1994, 1995a,1995b; Ueno & Suzuki, 2014).

The blue‐breasted quail originates from India, Southeast Asia, and the southeastern area of China and is sometimes reared as a pet (Uchida & Shimasaki, 1987; Yamashina, 1986). The blue‐breasted quail is the smallest species in the order Galliformes and is about half the body size of a Japanese quail, enabling easy handling. Moreover, this species exhibits excellent precocious and prolific properties under artificial rearing conditions, similar to the Japanese quail (Tsudzuki, 1994). Therefore, advantages of using the blue‐breasted quail as avian research model are that reduction in the cost, space and labor for breeding.

Currently, most of the methodologies used in avian developmental studies involve access to and manipulation of developing embryos. In chickens and Japanese quail, it is possible to culture an embryo ex vivo from the single‐cell stage, which normally develops in the oviduct, through to hatching (Naito, Nirasawa, & Oishi, 1990; Ono et al., 1994; Perry, 1988). Ex vivo culture enables easier access and precise manipulation of the developing avian embryos to produce chimeras by transplanting the blastoderm and primordial germ cells (Kagami et al., 1997; Nakamura, 2017; Nakamura et al., 2008, 2009, 2010; Tagami et al., 2007). The ex vivo culture system, from the freshly oviposited blastoderm stage through to hatching, has been applied to the blue‐breasted quail (Ono et al., 2005). Indeed, the blue‐breasted quail embryos could be manipulated as with the Japanese quail embryos due to the nearly the same size of embryos during early development (Y. Nakamura, Y. Nakane, M. Tsudzuki, unpublished data). Hence, the blue‐breasted quail may represent an alternative model organism in avian developmental studies.

To develop the blue‐breasted quail as an alternative avian model, it is desirable to describe its normal development in detail. A normal developmental series of the avian embryo was first described for chickens based on their gross morphology (Hamburger & Hamilton, 1951). This staging series has been the mainstay in avian developmental studies. Similarly, a definitive developmental staging series has been developed for Japanese quail that is focused on developmental features and feather pigmentation patterns (Ainsworth, Stanley, & Evans, 2010; Zacchei, 1961). These developmental series for chickens and Japanese quail are largely applicable to the development of blue‐breasted quail, although there might be some divergent developmental features among these species. Therefore, a definitive developmental staging series should be described for the blue‐breasted quail.

The purpose of this study was to document the normal staging table or the blue‐breasted quail based on its developmental features. Thereafter, comparative studies of the embryonic development of the blue‐breasted quail, chickens, and Japanese quail were conducted.

## 2 MATERIALS AND METHODS

### 2.1 Birds and eggs

A closed population of wild‐type, blue‐breasted quail that had been established from 10 pairs of birds originating from Taiwan (Tsudzuki, 1994) were used. Fertilized eggs were collected within 6 hr of laying and stored at 15°C. Fertilized eggs were used within 5 days of collection. All animal care and use in this study was conducted in accordance with Guidelines for the Proper Conduct of Animal Experiments (Science Council of Japan, 2006) and the animal experimentation guidelines of the Hiroshima University Animal Research Committee.

### 2.2 Preparation of embryo samples

Fertilized eggs were incubated (MIC‐14C; M's Factory, Nagoya, Japan) under the same conditions as Japanese quail (Nakane & Tsudzuki, 1999) at 37.7 ± 0.2°C and relative humidity of 70%, with rotation of 90º once every hour. The incubation period of the blue‐breasted quail in these conditions was approximately 16 days. For a description of the definitive developmental staging series, embryos were collected every hour for the first 45 hr, every 2 hr from 46 to 54 hr, every 3 hr from 57 to 72 hr, every 12 hr from 3.5 to 10 days, and every day from 11 to 16 days. Whole embryos were separated from the egg yolk, rinsed using phosphate‐buffered saline without Ca^2+^ or Mg^2+^ (PBS(‐)) and fixed in either Bouin's solution or 10% formalin.

### 2.3 Photography and measurement of embryos

The weight (mean ± *SD*) of each embryo at 3.5 days of development and older were measured immediately after sampling. Embryo images were taken before fixation using a Leica S9i stereomicroscope with an integrated camera (Leica Microsystems, Tokyo, Japan). For 6–16‐day embryos and newly hatched chicks, the length of the beak from the anterior angle of the nostril to the tip of the bill and the third toe length from tip to the middle of metatarsal (mean ± *SD*) of fixed embryos were measured using either a micrometer or a Vernier caliper under a stereomicroscope.

## 3 RESULTS

**Figure 2:**
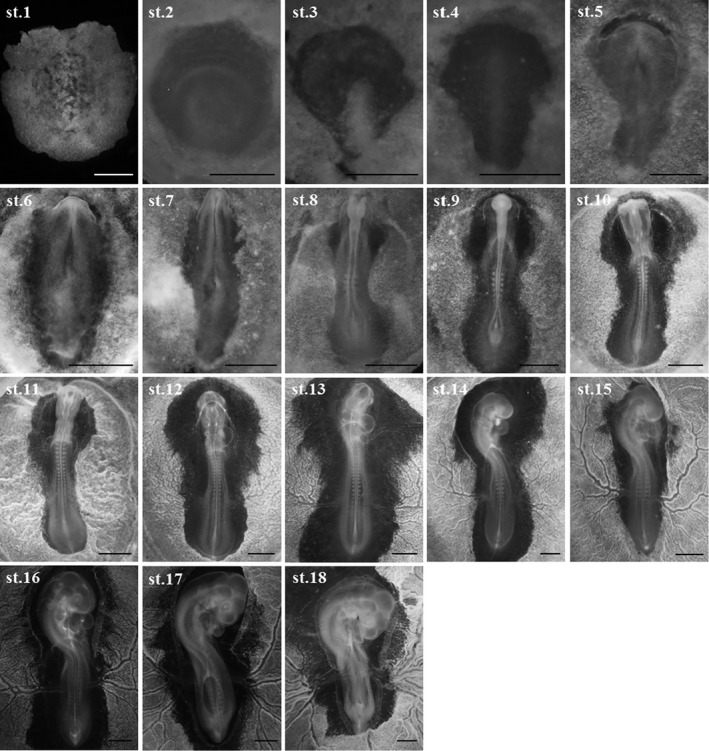
Images of blue‐breasted quail embryos at stages 1–18. Scale bars = 1 mm

**Figure 3:**
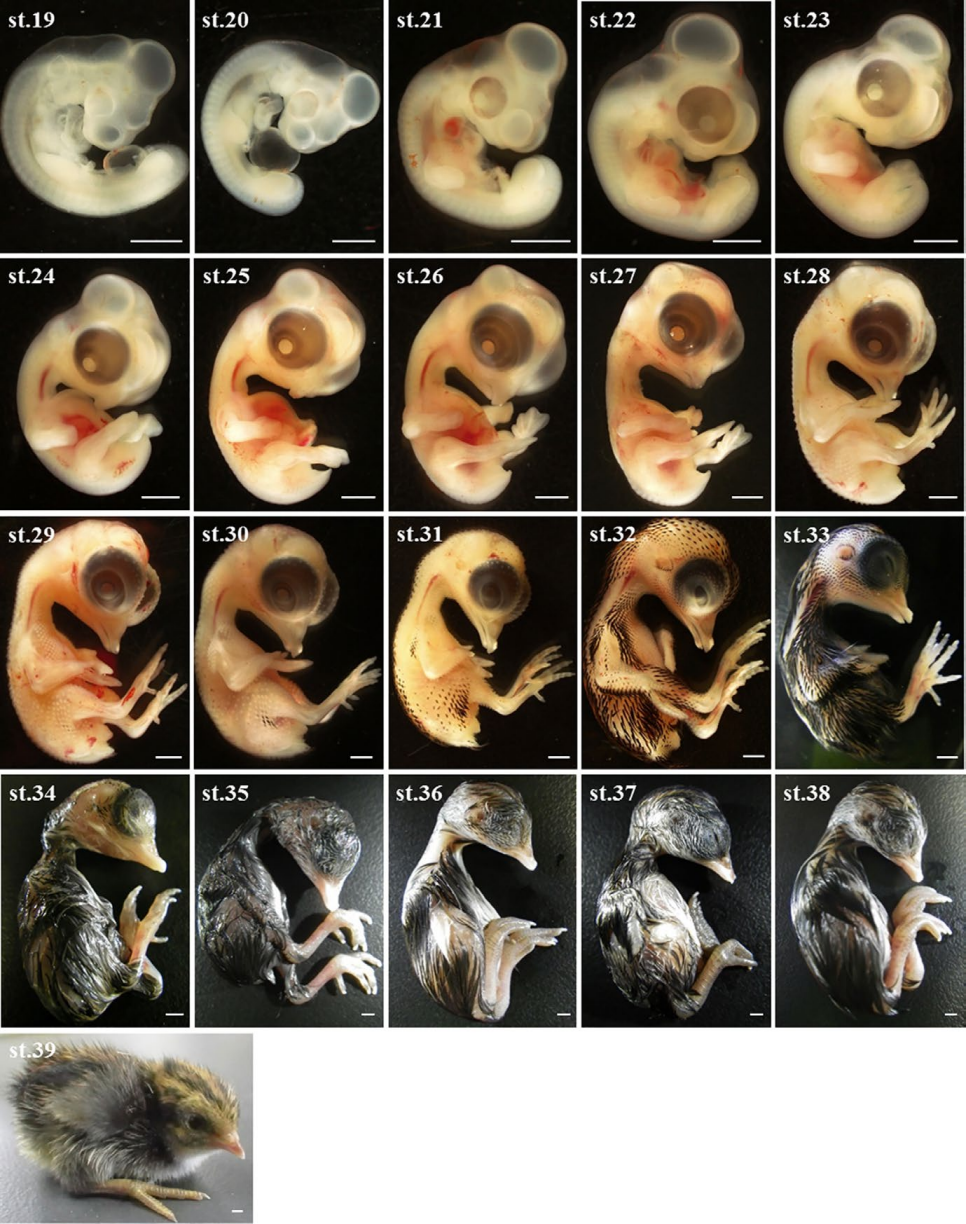
Images of blue‐breasted quail embryos at stages 19–38, and a newly hatched chick (stage 39). Scale bars = 2 mm

**Figure 4:**
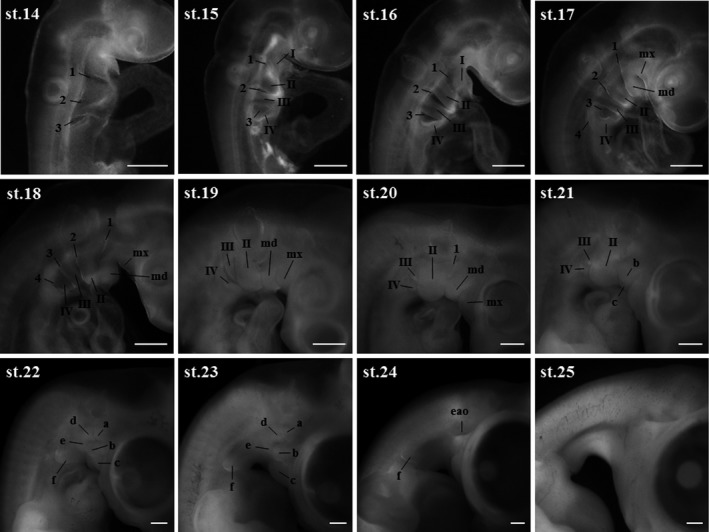
Images of visceral arches of blue‐breasted quail embryos at stages 14–25. 1–4, visceral clefts; I–IV, visceral arches; mx and md, maxillary and mandibular processes of visceral arch I; a–c, protuberances on the mandibular process; d–f, protuberances on visceral arch II; eao, external audio opening. Scale bars = 500 μm

**Figure 5:**
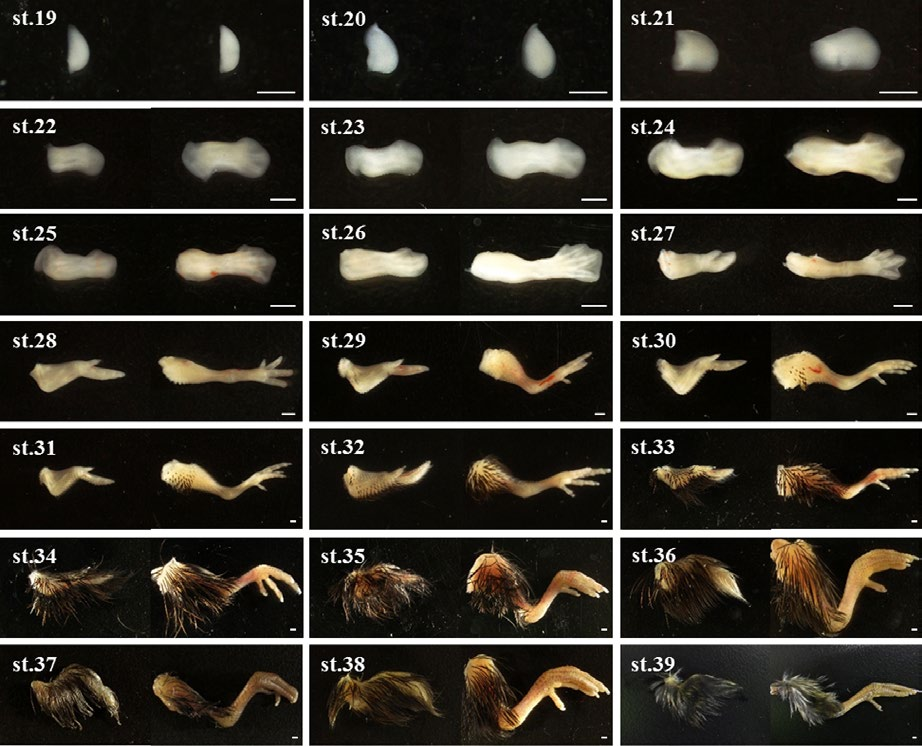
Images of limb buds of blue‐breasted quail embryos at stages 19–38, and a newly hatched chick (stage 39). Photographs representing wing‐ (left) and leg (right) buds. Scale bars = 1 mm

**Figure 6:**
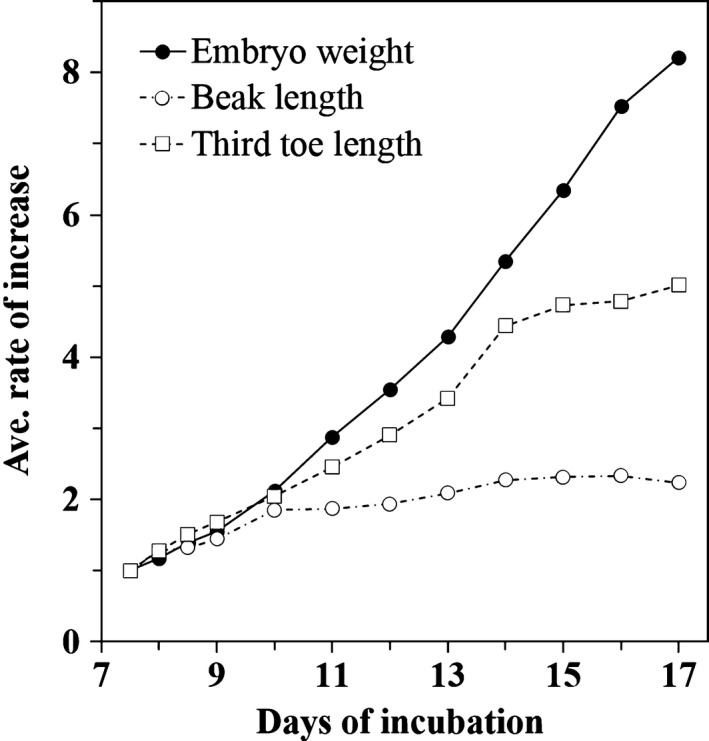
Average rate of increase in embryo weight, beak length, and third toe length on and after 7.5 days of incubation. Increase rates of embryo weight, beak length and third toe length were calculated by average measurements on each days dividing by that on day 7.5

Blue‐breasted quail embryos at 0–16 days of incubation and newly hatched chicks were staged based on changes in morphology (Figures 2 and 3). In this study, a series of 39 stages in total was documented (Table 1). Development of visceral arches and limb buds are shown in Figures 4 and 5, respectively. The weight of embryos at each developmental stage was expressed as mean ± *SD* on/after stage 20. The beak length and the third toe length at each developmental stage were presented as mean ± *SD* on/after stages 26 and 28, respectively. The average rate of increase in the beak length, third toe length, and embryo weight are shown in Figure 6. Feather germs appeared on/after stage 25. The distribution pattern of the feather germs and their pigmentation in each part of the body are summarized in Table 2.

**Table 1:** Summary of some key significant features of blue‐breasted quail development

Stage	Time of incubation	Significant key features
1	0–9 hr	No appearance of the primitive streak
2	6–11 hr	The primitive strak was visible
3	10–15 hr	The Hensen's node was present
4	12–17 hr	The primitive strak was fully elongated
5	16–21 hr	The head process was visible
6	20–24 hr	The head fold was apparent
7	22–26 hr	One somite was evident
8	25–28 hr	Four somites were evident
9	29–31 hr	Seven somites were evident
10	34–36 hr	10 somites were evident
11	37–40 hr	13 somites were evident
12	40–42 hr	16 somites were evident
13	43–45 hr	19 somites were evident
14	45–48 hr	22 somites were evident
15	50–52 hr	25 somites were evident
16	52–54 hr	26−27 somites were evident
17	57–60 hr	Limb buds were indicated
18	63–66 hr	The allantois appeared
19	69–72 hr	The amnion was completely closed
20	3.5 days	Faint eye pigmentation was visible
21	4 days	Limb buds were greater in length than width
22	4.5 days	Six protuberances were evident. Knee joints were indicated
23	5 days	The “Collar” was apparent
24	5.5 days	The nictitating membrane appeared. Beak outgrowth was apparent
25	6 days	Four scleral papillae were evident. The feather germs were visbile
26	6.5 days	14 scleral papillae were evident
27	7 days	The second digit and third toe were differentially grown
28	7.5 days	Brown pigmentation appeared in the dorsal chest‐lumbus region. Webs between the digits and toes were inconspicuous
29	8 days	Brown pigmentation extended to the thigh, scapula and ulna. Primodia of the claws indicated on the termini of all toes
30	8.5 days	Primodia of the claws appeared on the first digits
31	9 days	The scales appeared in the metatarsus and toe. Brown pigmentation extended to the crown
32	10 days	Appearance of the feather germs was mostly complete
33	11 days	The metatarsus and toes pigmented light‐gray. Feather pigmentation was visible
34	12 days	Embryo weight = 1.81. Beak length = 2.23
35	13 days	Embryo weight = 2.19. Beak length = 2.40
36	14 days	Embryo weight = 2.73. Beak length = 2.61
37	15 days	Embryo weight = 3.24. Beak length = 2.66
38	16 days	Embryo weight = 3.84. Beak length = 2.68
39	17 days	Hatching

**Table 2:** Distribution pattern of the feather germs and their pigmentation in blue‐breasted quail embryo

Parts of the body	Stages									
	25	26	27	28	29	30	31	32	33	34
Dorsal part										
Cervix	―	〇	〇	〇	●	●	●	●	●	●
Back	〇	〇	〇	●	●	●	●	●	●	●
Lumbus	〇	〇	〇	●	●	●	●	●	●	●
Cauda	―	―	―	〇	●	●	●	●	●	●
Ventral part										
Cervix	―	―	―	〇	〇	〇	〇	〇	〇	〇
Chest	―	〇	〇	〇	〇	●	●	●	●	●
Around umbilical cord	―	―	―	〇	〇	〇	〇	〇	〇	〇
Forelimb										
Brachium	―	―	〇	〇	〇	●	●	●	●	●
Alula	―	―	〇	〇	〇	〇	〇	●	●	●
Anterior edge of second digit	―	―	―	―	―	〇	〇	●	●	●
Posteior edge of third digit	―	―	―	―	〇	●	●	●	●	●
Hindlimb										
Thigh	―	〇	〇	〇	●	●	●	●	●	●
Tibiofibular region	―	―	―	―	―	〇	〇	●	●	●
Head part										
Forehead	―	―	―	〇	〇	〇	〇	●	●	●
Back of head	―	―	―	〇	〇	〇	〇	●	●	●
Crown	―	―	―	―	〇	〇	●	●	●	●
Bucca	―	―	―	―	〇	〇	〇	●	●	●
Around external auditory meatus	―	―	―	―	〇	〇	〇	●	●	●
Mandible	―	―	―	〇	〇	〇	〇	〇	〇	〇
Periocular region	―	―	―	〇	〇	〇	〇	●	●	●
Around nasal pit	―	―	―	―	―	―	―	〇	〇	●

Note. ―, Not observed; 〇, Presence of feather germs without pigmentation; ●, Presence of pigmented feather germs.

Key morphological features of blue‐breasted quail development at each stage were summarized in Table 1. One of the primary developmental events at stages 1–6 was the formation of the primitive streak and notochord. Hence, the degrees of primitive streak formation and notochord formation can be used as criterion in these stages. At stages 7–16, one of the significant developmental events was somitogenesis. Thus, somite number can be used to define these stages. At stages 17–24, one of the remarkable developmental events was limb bud formation. For these stage, the degrees of limb buds combined with other key morphological features as shown in Table 1 can be used for staging. At stages 25–33, feather germ formation and their pigmentation were one of the conspicuous developmental events. Therefore, blue‐breasted quail embryos can be staged based on the distribution and pigmentation patterns of feather germs. At stages 34–39, most of the external features have lost their diagnostic value due to few morphological change. Embryo weight and third toe length can be used as reliable criterion in these stages. The detailed morphological features at each developmental stage were described below.

### 3.1 Stage 1: Pre‐streak (0–9 hr)

The blastoderm was composed of two layers, an upper layer (epiblast) and a lower layer (hypoblast). The inner area pellucida and peripheral area opaca were in the form of concentric circles. The primitive streak had not yet appeared.

### 3.2 Stage 2: Initial primitive streak (6–11 hr)

The rudimentary primitive streak had a rope‐shaped thickening that extended from the posterior border to approximately the center of the pellucida area. The streak somewhat broadened where it touched the area opaca.

### 3.3 Stage 3: Intermediate primitive streak (10–15 hr)

The Hensen's node was present at the anterior end of the primitive streak. The area pellucida had become pear‐shaped and the primitive streak extended approximately two‐thirds of its length. The streak was clearly narrow throughout its length compared to that in stage 2.

### 3.4 Stage 4: Definitive primitive streak (12–17 hr)

The primitive groove and primitive pit were recognizable. The primitive streak had reached its maximum length and extended approximately three‐quarters the length of the area pellucida.

### 3.5 Stage 5: Head process (16–21 hr)

The head process was visible as a condensed rope‐shape that extended anterior to the Hensen's node. First indication of the head fold appeared as a bulge, which marked the anterior border of the embryo anterior to the head process. The posterior end of the streak was detached from the boarder of the area opaca.

### 3.6 Stage 6: Head fold (20–24 hr)

The neural plate and neural groove were present. A definitive head fold extended as a fold in the anterior part of the embryo.

### 3.7 Stage 7: One somite (22–26 hr)

One somite appeared at approximately the center of the embryo body where it was located anterior to the Hensen's node. This somite was equivalent to the second somite of the series. The first somite was not yet clearly defined. The neural folds were visible as bulges on each side of the neural groove. The head fold extended like a semi‐dome type structure.

### 3.8 Stage 8: Four somites (25–28 hr)

Four somites were visible. The primordia of the optic vesicles appeared at the anterior end of the neural folds. The area pellucida became elliptical‐shaped.

### 3.9 Stage 9: Seven somites (29–31 hr)

Seven somites were present. Both neural folds were fused from the region of the optic vesicles to the lateral end of the brain and formed the neural tube. Paired primordia of the heart began to fuse. Blood islands were scattered in a semi‐circle in the posterior part of the area opaca adjacent to the border of the area pellucida.

### 3.10 Stage 10: Ten somites (34–36 hr)

Ten somites were present, but the first somite was beginning to disappear. This somite was not included in the counts for subsequent stages. The neural folds had completely closed, except for a rhombus‐shaped hole in the posterior part. Three primary brain vesicles (forebrain/midbrain/hindbrain) were distinct. The heart bent slightly to the right. Blood islands increased in number. The area pellucida had become gourd‐like in shape.

### 3.11 Stage 11: Thirteen somites (37–40 hr)

Thirteen somites were present. The neural folds were fused from the anterior end to the posterior end of the embryo, but the anterior neuropore was still opening. The rhombomeres divided the hindbrain into seven smaller compartments. The heart made a bold curve and protruded to the right side. Cranial flexure was slightly indicated. Blood islands were distinct in the lateral parts of the area opaca. Auditory pits were seen at both sides of the embryo body at the posterior end of the myelencephalon. The optic vesicles were constricted at their bases.

### 3.12 Stage 12: Sixteen somites (40–42 hr)

Sixteen somites were present. Anterior neuropore was closed. Five secondary vesicles (telencephalon/diencephalon/mesensephalon/metencephalon/myelencephalon) were indicated. Blood islands were fused with each other and blood networks were visible. A peripheral vein was indicated. Head was turned to the right. The optic stalk was well established. The heart showed an S‐shape and the atrio‐ventricular canal was indicated by constriction.

### 3.13 Stage 13: Nineteen somites (43–45 hr)

Nineteen somites were present. The body was rotated back as far as somites 7–9. The head was partially turned to the right. Cranial and cervical flexures made a broad curve. The lens placode had formed. The head fold of the amnion was covered anteriorly to somite 2.

### 3.14 Stage 14: Twenty‐two somites (45–48 hr)

Twenty‐two somites were present.

*Flexures and rotation*. Rotation extended from somites 10–12. The head was fully turned to the right. Cranial flexure was approximately right‐angled between the axes of the forebrain and hind brain. Cervical flexure made a broad curve.

*Amnion*. The amnion extended from somites 2–7.

*Visceral arches*. Visceral clefts 1, 2, and 3 were indentified.

### 3.15 Stage 15: Twenty‐five somites (50–52 hr)

Twenty‐five somites were present.

*Flexures and rotation*. Cranial flexure was an acute angle between the axes of the forebrain and hind brain. The cervical flexure was nearly 90°.

*Visceral arches*. Visceral clefts 1 and 2 were a narrow groove, but visceral cleft 3 remained oval‐shaped. Visceral arches I, II, III, and IV were distinct. The elencephalon was developing.

*Lateral body fold*. The lateral body folds were indicated.

### 3.16 Stage 16: Twenty‐six to twenty‐seven somites (52–54 hr)

Twenty‐six to twenty‐seven somites were present.

*Flexures and rotation*. Cranial and cervical flexures were more accentuated than in the previous stage. The anterior tip of the telencephalon was level with somites 4–6.

*Amnion*. The amnion extended from somites 11–14.

*Visceral arches*. Visceral cleft 3 enlarged, but remained shorter than cleft 2.

*Lateral body fold*. The lateral body fold extended to around somite 19.

*Tail bud*. The tail bud was represented as a condensation at the posterior tip of the embryo.

Beyond stage 16, the number of somites became difficult to accurately measure owing to the dispersion of their anterior parts, developing tail bud, and trunk flexure.

### 3.17 Stage 17: (57–60 hr)

*Flexures and rotation*. Cranial and cervical flexures were more sharply bent than in preceding stages. The anterior tip of the telencephalon was level with somites 11–13.

*Amnion*. The amnion covered the entire body, except for an oval‐shaped hole in the lumber region.

*Visceral arches*. Visceral arches I and II clearly enlarged. Visceral cleft 4 was indicated.

*Lateral body fold*. The lateral body fold extended around the entire circumference of the body.

*Tail bud*. The tail bud was enlarged and bent slightly to the left.

*Limb buds*. Both wing and leg buds were represented by thickened ridges.

### 3.18 Stage 18: (63–66 hr)

*Flexures and rotation*. Cervical flexure formed an acute angle between the axes of the medulla and the posterior trunk. Rotation was apparent at the posterior end of the body. The anterior end of the diencephalon was level with the wing buds.

*Amnion*. The amnion showed complete closure, except for a small hole.

*Visceral arches*. Visceral cleft 4 was distinct.

*Tail bud*. The tail bud was turned to the left.

*Limb buds*. Both wing and leg buds were enlarged. L/W (L: length is the anterior‐posterior dimension as measured along the body wall; W: width is the distance from the body wall to the apex; see Hamburger & Hamilton, 1951) of wing and leg buds was 5 and 4, respectively.

*Epiphysis*. The epiphysis was indicated as a knob.

*Allantois*. An anlage of the allantois was indicated as a ridge between both leg buds.

### 3.19 Stage 19: (69–72 hr)

*Flexures and rotation*. The entire body had rotated, and the contour of the posterior part of the body was straight to the base of the tail. Cervical flexure was maximum. The anterior tip of the head was level with the leg buds.

*Amnion*. The amnion was completely closed.

*Visceral arches*. Both maxillary and mandibular processes were distinct in visceral arch I.

*Tail bud*. The tail bud was curved with the tip pointing forward.

L/W of wing and leg buds was 3.5 and 3, respectively.

*Allantois*. The allantois was represented as a thick‐walled capsula.

### 3.20 Stage 20: (3.5 days)

*Visceral arches*. Visceral arches I and II were enlarged. The mandibular process in visceral arches I and II were the same length. Visceral cleft 1 was visible as a slit as the mandibular process and visceral arch II had fused at their tips. The oral aperture, which met the nasal pits, was deep, but wide open.

*Limb buds*. Leg buds were apparently larger than wing buds from this stage. L/W of wing and leg buds was 1 and 0.8, respectively.

*Allantois*. The allantois had become thin, vesicular, and extended to the head.

*Eyes*. Eye pigmentation was indicated partly as a faint grayish hue.

Average weight of embryos was 30 ± 5 mg (*n* = 16).

### 3.21 Stage 21: (4 days)

*Visceral arches*. Visceral arch II extended over the surface and overlapped with visceral arch III ventrally. Two protuberances (“b” and “c”) appeared on the mandibular process. The groove was visible between protuberances “b” and “c”.

*Limb buds*. Wing limbs were bent slightly at the elbow joint. The toe plate in the leg buds was formed. Both wing and leg buds were distinctly longer than wide. L/W of wing and leg buds was 0.5 and 0.4, respectively.

*Epiphysis*. The epiphysis was indistinct.

*Eyes*. Whole circumferences of the eyes were darkened with pigment.

Average weight of embryos was 50 ± *10* mg (*n* = 15).

### 3.22 Stage 22: (4.5 days)

*Visceral arches*. Three protuberances (“a,” “b,” and “c”; from dorsal to ventral) on the mandibular process and three other protuberances (“e,” “f,” and “g”; from dorsal to ventral) on visceral arch II were distinct. Visceral arch II was enlarged ventrally at protuberance “f,” which was conspicuous and projected distinctly over the surface. Visceral arch III was obscured by visceral arch II. Protuberances “a,” “b,” “d,” and “e” were located around the audio pit. The maxillary process was lengthened and twice as long as the mandibular process. Narrowing of slit openings to deep nasal pits.

*Limb buds*. The digital plate in wings was formed, but no demarcation of the digits was observed. Faint grooves were indicated between the second, third, and fourth toes. Knee joints were indicated.

Average weight of embryos was 90 ± 10 mg (*n* = 15).

### 3.23 Stage 23 (5 days)

*Visceral arches*. Protuberance “f” had broadened backward to form the “collar.” The nasal pits had narrowed to a slit in the presumptive beak.

*Limb buds*. Grooves between the first, second, and third digits on the wings were distinct. The second to fourth toes on the legs stood out as ridges separated by distinct grooves with indications of webs between them.

*Eyes*. The anlage of the eyelids was indicated in the periphery of the eyes.

Average weight of embryos was 120 ± 20 mg (*n* = 10).

### 3.24 Stage 24: (5.5 days)

*Visceral arches*. The neck between the mandible process and “collar” had lengthened conspicuously. The “collar” was flattened. The protuberances “a” and “d”, and “b” and “e” had fused with each other. The external audio opening was distinct between these protuberances.

*Limb buds*. The second and third digits on the wings had lengthened. The contour of the wings was angular in the region of the first digit. Webs between the first and second toes on the legs were indicated.

*Eyes*. The nictitating membrane was indicated inside the eyelid.

*Beak*. The maxillary process had fused with the presumptive beak. The nasal pits had separated from the oral aperture.

Average weight of embryos was 200 ± 20 mg (*n* = 21).

### 3.25 Stage 25: (6 days)

*Visceral arches*. All protuberances “a,” “b,” “d,” and “e” had fused and flattened. The “collar” was recognized as a groove that formed a boundary between the neck and the breast.

*Limb buds*. The first digit on the wings was distinctive due to the conspicuously lengthened second digit.

*Eyes*. Approximately four scleral papillae had appeared on the dorsal side near the choroid fissure.

*Feather germs*. One dorsal row of feather germs was indicated along either side of the spine from the neck to the chest. In addition, one dorsal row on the spine had appeared in the lumbus.

*Beak*. The beak was more pronounced than in the former stage. The egg tooth was indicated.

Average weight of embryos was 270 ± 30 mg (*n* = 19).

### 3.26 Stage 26: (6.5 days)

*Visceral arches*. The external audio opening was mortar‐shaped. The posterior opening of the nostril was protuberated.

*Limb buds*. Wings were bent slightly in the carpal region. The contour of the web between the first and second digits was concave. Webs between all the toes were slightly curved and concave. Legs were distinctly bent at the knee joint.

*Eyes*. The nictitating membrane extended approximately one‐third the length between the eyelid and scleral papillae. Fourteen round‐shaped scleral papillae were present. The anterior tip of the mandible reached the beak.

*Feather germs*. The number of feather germs increased along the dorsal surface from the neck to the lumbus. Distinct feather germs appeared on the thigh and in the region overlaying the coracoids.

*Beak*. The anterior tip of the mandible reached the beak. The egg tooth was distinct and protruded.

Average weight of embryos was 340 ± 20 mg (*n* = 21).

Average length of beaks was 0.68 ± 0.08 mm (*n* = 20).

### 3.27 Stage 27: (7 days)

*Limb buds*. Differential growth of the second digit and third toe was conspicuous. Webs between the digits and toes were recessed. The legs were bent slightly at the ankle.

*Eyes*. The nictitating membrane extended halfway between the eyelid and scleral papillae.

*Feather germs*. One and three rows of feather germs were indicated for the first time anteriorly along the outer side of each eye and on the posterior edge of the brachium, respectively. The dorsal feather germs at the lumbus were enlarged into a fine hill shape.

Average weight of embryos was 420 ± 30 mg (*n* = 11).

Average length of beaks was 0.89 ± 0.11 mm (*n* = 10).

### 3.28 Stage 28: (7.5 days)

*Limb buds*. Webs between the digits and toes were inconspicuous. Differences in size of the first digit and individual toes were conspicuous. Distal segments of the leg were indicated as ridges. The second digit and third toe were lengthened. The third toe was one‐and‐a‐half times longer than the second toe.

*Eyes*. The nictitating membrane extended approximately three‐quarters the length of the eyelid and scleral papillae. The eyelids extended towards the beak, and their circumference was ellipsoidal.

*Feather germs*. Feather germs appeared on the tail, knee, alula, lower eyelid, ventral surface of the neck, mandible, forehead, back of the head, and around the umbilical cord. Eleven rows of feather germs were present between the eyes. Feather germs on the thigh and along the dorsal surface from the chest to the lumbus were conspicuous and conical. Brown pigment appeared at the tip of each feather germ in one dorsal row along either side of the spine from the chest to the lumbus and one dorsal row along the spine in the region of the pelvis.

Average weight of embryos was 510 ± 30 mg (*n* = 15).

Average length of beaks was 1.15 ± 0.09 mm (*n* = 14).

Average length of third toes was 2.14 ± 0.12 mm (*n* = 14).

### 3.29 Stage 29: (8 days)

*Limb buds*. The tapering primordia of the claws appeared on the termini of all toes.

*Eyes*. The nictitating membrane covered the anterior‐most scleral papillae. Circumferences of eyelids were narrowing ellipses.

*Feather germs*. New feather germs appeared in the buccal region, adjacent to the external auditory meatus, the posterior edge of the third digit, and parietal region. The feather germs were prominent on the brachium, thigh, and dorsal surface from the back of the head to the lumbus, and these regions were merged. Brown color pigmentation was newly apparent in the feather germs on the lateral aspects of the thigh and overlaying the scapula and ulna.

Average weight of embryos was 600 ± 30 mg (*n* = 13).

Average length of beaks was 1.47 ± 0.15 mm (*n* = 13).

Average length of third toes was 2.72 ± 0.20 mm (*n* = 13).

### 3.30 Stage 30: (8.5 days)

*Limb buds*. Pads on the plantar surface of the foot were indicated. Primordia of the claws of all the toes were lengthened. New primordia of the claws appeared on the first digits of each wing.

*Eyes*. The nictitating membrane approached the cornea.

*Feather germs*. Newly appeared feather germs were visible on the tibiofibular region and the anterior edge of the second digit. Black pigmented feather germs were more numerous on the dorsal surface, thigh, and overlaying the scapula and ulna. Dark reddish‐brown pigmentation was newly recognizable in the feather germs on the ventral surface, brachium, and part of the posterior edge of the third digit. Feather germs in the most advanced regions, for example, along the back and on the tail, were elongated into long and sharply tapered cones. Two rows of feather germs with light‐brown pigment were also apparent on the ventral surface.

Average weight of embryos was 710 ± 40 mg (*n* = 16).

Average length of beaks was 1.52 ± 0.10 mm (*n* = 16).

Average length of third toes was 3.23 ± 0.16 mm (*n* = 16).

### 3.31 Stage 31: (9 days)

*Limb buds*. First indication of scales appeared as rugged skin on the superior surfaces of the metatarsus and the plantar surface of toes.

*Eyes*. Circumference of the eyelids presented as a teardrop‐shape. Lower eyelids extended upward to the cornea.

*Feather germs*. One row of feather germs along either side of the midline of the crown was newly pigmented. Primordia of the claws of all the toes were longer than at stage 30. The feather germs along the dorsal surface of the back, on the lateral aspects of the thigh, and on the tail were enlarged.

Average weight of embryos was 790 ± 50 mg (*n* = 13).

Average length of beaks was 1.66 ± 0.09 mm (*n* = 10).

Average length of third toes was 3.60 ± 0.12 mm (*n* = 10).

### 3.32 Stage 32: (10 days)

*Limb buds*. Scale primordia on the superior surfaces of the metatarsus and the plantar surface of the toes had grown and were slightly protuberated. Scale primordia were indicated on the inferior surfaces of the metatarsus.

*Eyes*. Upper eyelids reached the dorsal edge of the cornea and lower eyelids covered one‐half of the cornea.

*Feather germs*. Feather germs appeared between the nasal pit and upper jaw. Circumference of the eyelids was bordered by a single row of primordia. Appearance of the feather germs was mostly complete at this stage. Pigmentation of the feather germs was observed in most of the planned pigmentation areas, except for the buccal region, between the nasal pit and upper jaw, and along the anterior edge of the wing. Pigmentation of the feathers was not yet indicated.

Average weight of embryos was 1.08 ± 0.07 g (*n* = 20).

Average length of beaks was 2.13 ± 0.10 mm (*n* = 19).

Average length of third toes was 4.38 ± 0.19 mm (*n* = 19).

### 3.33 Stage 33: (11 days)

*Limb buds*. Primordia of the scales on the superior surfaces of the metatarsus and the plantar surface of the toes were overlapping and pigmented light‐gray.

*Feather germs*. Pigmentation was observed within the periocular region. The length of the feather germs had greatly increased in major tracts. In particular, feather germs on the dorsal surface, thigh, and tail had emerged similar to that observed in newly hatched chicks. Feather pigmentation was indicated for the first time in the feather germs that appeared in the earlier stages.

Average weight of embryos was 1.47 ± 0.09 g (*n* = 16).

Average length of beaks was 2.15 ± 0.16 mm (*n* = 16).

Average length of third toes was 5.25 ± 0.17 mm (*n* = 16).

### 3.34 Stage 34: (12 days)

*Limb buds*. Pigmentation in the scales thickened.

*Eyes*. Opening between the eyelids was reduced to a thin crack.

*Feather germs*. Feather germs were lengthened in major tracts, particularly on the crown. Pigmented feather germs appeared between the nasal pit and upper joe. At this stage, pigmentation of the feather germs was recognizable in all the planned pigmentation areas.

*Beak*. Beak was thicker at the tip. Light‐pink pigmentation was distinct in the egg‐tooth.

Average weight of embryos was 1.81 ± 0.11 g (*n* = 18).

Average length of beaks was 2.23 ± 0.12 mm (*n* = 18).

Average length of third toes was 6.22 ± 0.24 mm (*n* = 18).

Beyond stage 34, external features lost their diagnostic value. Stages 35 to 39 were based mainly on the weight of embryos, the length of beaks, and the length of third toes. Of these three criteria, the weight of embryos was the most informative, as there were relatively large differences among stages (Figure 6).

### 3.35 Stage 35: (13 days)

*Limb buds*. Pigmentation on the metatarsus and toes thickened partly in the shape of a belt. The entire plantar surface of the phalanges was covered with well‐grown papillae. The toes were thickening.

*Eyes*. The eyelids were closing. Black pigmented feather germs were prominent within the periocular region.

Average weight of embryos was 2.19 ± 0.15 g (*n* = 21).

Average length of beaks was 2.40 ± 0.11 mm (*n* = 21).

Average length of third toes was 7.32 ± 0.27 mm (*n* = 21).

### 3.36 Stage 36: (14 days)

Length of the neossoptile increased. Upper bill and scales on the legs were pigmented and thicker.

Average weight of embryos was 2.73 ± 0.21 g (*n* = 20).

Average length of beaks was 2.61 ± 0.11 mm (*n* = 18).

Average length of third toes was 9.50 ± 0.42 mm (*n* = 18).

### 3.37 Stage 37: (15 days)

Upper bill and circumference of the nostrils was pigmented with yellowish‐brown and light‐brown, respectively. One‐third of the yolk‐sac was enclosed within the body cavity. Hence, the umbilical ring was slightly larger than at stage 36.

Average weights of embryos with and without the yolk‐sac were 4.39 ± 0.34 g (*n* = 18) and 3.24 ± 0.30 g (*n* = 16), respectively.

Average length of beaks was 2.66 ± 0.12 mm (*n* = 18).

Average length of third toes was 10.12 ± 0.51 mm (*n* = 18).

### 3.38 Stage 38: (16 days)

Enclosure of the yolk‐sac within the body cavity was nearly complete, but the umbilical ring had not yet closed. Upper bill was brown in color. The chorioallantoic membrane contained less blood and was sticky in an inner eggshell.

Average weights of embryos with and without the yolk‐sac were 4.47 ± 0.40 g (*n* = 20) and 3.84 ± 0.27 g (*n* = 16), respectively.

Average length of beaks was 2.68 ± 0.15 mm (*n* = 20).

Average length of third toes was 10.24 ± 0.37 mm (*n* = 20).

### 3.39 Stage 39: (17 days)

Newly hatched chick. Outer layer of the neossoptile was detached and indicated as a napped shape. Closure of the umbilical ring was complete. Inner eggshell was dry.

Average weight of chicks was 4.19 ± 0.15 g (*n* = 23).

Average length of beaks was 2.57 ± 0.21 mm (*n* = 23).

Average length of third toes was 10.73 ± 0.45 mm (*n* = 23).

## 4 DISCUSSION

The Hamburger and Hamilton (1951) staging series for chicken embryos has been most widely used as a normal table for staging avian embryos in various species. This staging table is roughly applicable to other avian species, for example, Japanese quail, turkey, duck, goose, and guinea fowl in the first 72 hr of incubation, although there are slight developmental differences (Sellier, Brillard, Dupuy, & Bakst, 2006). Moreover, developmental heterochrony was observed between chickens and Japanese quail, as well as between chickens and emu (*Dromaius novaehollandiae*) (Ainsworth et al., 2010; Nagai et al., 2011). Therefore, a comparative study of normal development was conducted between blue‐breasted quail, chickens, and Japanese quail based on the developmental series in this study (NNT). The developmental differences and heterochrony between these three species are as follows.

To compare the external appearance and developmental heterochrony between the three species, the Hamburger and Hamilton (1951) staging table for chickens (HH) and the Ainsworth et al. (2010) staging table for Japanese quail (ASE) were referenced.

### 4.1 Stages 1–6

Analysis of the blue‐breasted quail revealed that the order and time of appearance of the primitive streak, primitive groove, primitive pit, head process, head fold, neural plate, and neural groove were equivalent in chickens and Japanese quail. Thus, NNT1–6 of blue‐breasted quail embryos can be compared directly with chicken and Japanese quail embryos.

### 4.2 Stages 7–16

The most conspicuous criterion for this phase of development was the number of pairs of somites. Thus, somite numbers were compared based on developmental features. The optic vesicle appeared earlier in blue‐breasted quail than in chickens. Namely, the optic vesicle observed in seven somites stage (HH9) chicken embryos was observed at the four somites stage (NNT8) in blue‐breasted quail embryos. Conversely, the neural folds fused later in blue‐breasted quail than in chickens, which occurred at the seven somites stage (NNT9) in blue‐breasted quail and at the four somites stage (HH8) in chickens. The timing of appearance of the blood island was variable among the three species, at the seven somite stage (NNT9) in blue‐breasted quail, at the four somites stage (HH8) in chickens, and at the one somite stage (ASE7) in Japanese quail. The rate of blue‐breasted quail development in these stages was nearly identical to that of chickens and Japanese quail.

### 4.3 Stages 17–24

Development of the limb bud is a commonly used criterion in these stages of avian embryos. Hence, the degrees of limb bud development were compared based on other externally visible features. Limb bud formation started at approximately the same period of development in blue‐breasted quail (NNT17; 57–60 hr of incubation), chickens (HH16–17; 51–64 hr of incubation), and Japanese quail (ASE16–17; 51–64 hr of incubation). Eye pigmentation also occurred at an equivalent developmental stage in the three species (NNT20, HH20, and ASE20; 3.5 days of incubation). However, limb bud morphology in NNT20 blue‐breasted quail embryos corresponded to that of HH23 (3.5–4 days of incubation) in chicken embryos and ASE23 (4 days of incubation) in Japanese quail embryos. The scleral papillae and egg tooth appeared at NNT25 (6 days of incubation) in blue‐breasted quail embryos, with overall external features similar to HH30 (6.5 days of incubation) chicken embryos and ASE30 (6–6.5 days of incubation) Japanese quail embryos. However, morphological features of the limb buds of blue‐breasted quail at this stage corresponded to those of chicken embryos at HH31 (7 days of incubation) and Japanese quail at ASE29 (5.5–6 days of incubation). Therefore, the early phase of limb bud growth was fastest in blue‐breasted quail, but subsequent limb growth was fastest in Japanese quail.

Developmental heterochrony in morphological changes of protuberances “a” to “f” on the second visceral arch was observed between the three species. Indications of all protuberances were later in blue‐breasted quail and chickens (4.5 days of incubation) than in Japanese quail (4 days of incubation). Hereafter, retarded morphology of the protuberances in blue‐breasted quail and chickens continued in the same period of incubation. Disappearance of protuberance structure was earlier in both species of quail (6 days of incubation) than in chickens (7 days of incubation).

### 4.4 Stages 25–33

This stage of chicken embryos was based on the pattern of feather germs and their degree of growth (Hamburger & Hamilton, 1951). In addition, feather pigmentation pattern was primarily used for staging Japanese quail embryos (Ainsworth et al., 2010). Though a simple comparison was difficult, the degree of limb bud growth was compared based on other developmental features, for example, the growth of the nictitating membrane and the eyelids. A developmental stage in which the nictitating membrane covered half the length between the eyelid and the scleral papillae corresponded to NNT27 (7 days of incubation) in blue‐breasted quail, HH34 (8 days of incubation) in chickens, and ASE34 (7.5 days of incubation) in Japanese quail. However, developmental features of the limb buds at NNT31 in blue‐breasted quail embryos were equivalent to those of HH35 (8 days of incubation) chicken embryos and ASE32–33 (7 days of incubation) Japanese quail embryos. NNT31 (9 days of incubation) blue‐breasted quail embryos, whose lower eyelid had grown upward to the level of the cornea, corresponded to HH34 (8 days of incubation) chicken embryos and ASE34 (7.5 days of incubation) Japanese quail embryos. However, limb bud morphology of blue‐breasted quail embryos at this stage was equivalent to that of HH37 (11 days of incubation) chicken embryos and ASE36–38 (9 days of incubation) Japanese quail embryos. In this stage of development, limb bud growth in blue‐breasted quail was earlier than that in chickens, but later than that in Japanese quail.

After 6.5 days of incubation (NNT26), there was a gradual acceleration in the rate of blue‐breasted quail ontogeny compared with that in chickens. Though the developmental rate of embryos was almost the same between the two quails, there were slight differences in the timing of appearance of the primordia of the claws and feather germs. Primordia of the claws were recognizable earlier in blue‐breasted quail (8.5 days of incubation) than in Japanese quail (9 days of incubation). Indication of feather germs was also earlier in blue‐breasted quail (6 days of incubation) than in Japanese quail (6.5 days of incubation).

### 4.5 Stages 34–39

Though a major criterion of this stage of development was the lengths of the beaks and third toes, these measurements were not useful for direct comparisons between blue‐breasted quail, chickens, and Japanese quail due large differences in their body sizes. Nevertheless, species‐specific morphological features were available for staging, for example, pigmentation of the feather germs in the two quails and growth of the comb in chickens. Feather germ pigmentation started earlier in blue‐breasted quail (7 days of incubation) than in Japanese quail (8–9 days of incubation). However, after 7 days of incubation, the incubation time and order of pigmentation was mostly similar in the two quails.

In this paper, we described a definitive normal staging table for the blue‐breasted quail, including specific developmental features after specific periods of incubation. Blue‐breasted quail embryos can be directly compared with chicken and Japanese quail embryos at earlier stages of development (stages 1–16) using the HH staging table; however, NNT staging table is especially useful at later stages of development (stages 34–39) of blue‐breasted quail. The developmental staging table of blue‐breasted quail described here will be useful as a normal control in the fields of general embryology, avian developmental biology as well as in the study of mutants.
